# Measuring social networks in British primary schools through scientific engagement

**DOI:** 10.1098/rspb.2010.1807

**Published:** 2010-11-03

**Authors:** A. J. K. Conlan, K. T. D. Eames, J. A. Gage, J. C. von Kirchbach, J. V. Ross, R. A. Saenz, J. R. Gog

**Affiliations:** 1 CIDC, Department of Veterinary Medicine, University of Cambridge, Madingley Road, Cambridge CB3 0ES, UK; 2 Infectious Diseases Epidemiology Unit, London School of Hygiene and Tropical Medicine, Keppel Street, London WC1E 7HT, UK; 3 Millennium Mathematics Project, University of Cambridge, Centre for Mathematical Sciences, Wilberforce Road, Cambridge CB3 0WA, UK; 4 Department of Applied Mathematics and Theoretical Physics, University of Cambridge, Centre for Mathematical Sciences, Wilberforce Road, Cambridge CB3 0WA, UK; 5 School of Mathematical Sciences, North Terrace Campus, The University of Adelaide, Adelaide, SA 5005, Australia

**Keywords:** schools, social networks, scientific outreach, epidemiology

## Abstract

Primary schools constitute a key risk group for the transmission of infectious diseases, concentrating great numbers of immunologically naive individuals at high densities. Despite this, very little is known about the social patterns of mixing within a school, which are likely to contribute to disease transmission. In this study, we present a novel approach where scientific engagement was used as a tool to access school populations and measure social networks between young (4–11 years) children. By embedding our research project within enrichment activities to older secondary school (13–15) children, we could exploit the existing links between schools to achieve a high response rate for our study population (around 90% in most schools). Social contacts of primary school children were measured through self-reporting based on a questionnaire design, and analysed using the techniques of social network analysis. We find evidence of marked social structure and gender assortativity within and between classrooms in the same school. These patterns have been previously reported in smaller studies, but to our knowledge no study has attempted to exhaustively sample entire school populations. Our innovative approach facilitates access to a vitally important (but difficult to sample) epidemiological sub-group. It provides a model whereby scientific communication can be used to enhance, rather than merely complement, the outcomes of research.

## 1. Introduction

Schools are widely acknowledged to play an important role in the transmission of infectious diseases within human societies [1], evidenced by the association between the reproductive ratio of epidemic spread and the timing of school closures [2,3]. Despite this, surprisingly little quantitative data have been gathered on the social patterns of mixing within a school. In particular, no studies have attempted to gather network measures that are likely to be associated with the transmission of close contact infections such as measles, chicken pox and influenza from primary-school-aged children. Theoretical models for disease transmission based on divergent, even antithetical, assumptions concerning the heterogeneity of social contacts can nonetheless produce similar disease dynamics and equally well describe the available sources of epidemiological data [4]. However, the predictions of such models about the impact of control measures such as vaccination can be as divergent as their underlying assumptions [3,5–7]. Through quantifying the patterns of social mixing within schools, we set out to provide an empirical base for the refinement of mechanistic transmission models, ultimately seeking to improve predictions for assessing potential control measures on epidemics of novel pathogens.

In recent years, questionnaire-based methods have achieved great success in quantifying the social patterns of mixing pertinent to the spread of infectious diseases [8,9]. The structure of children's social networks have also received a great deal of interest in the social sciences [10–14], particularly with respect to the influence of peer networks on educational achievement [15]. However, studies have had limited success in obtaining information about school-age children, having either small sample sizes or low response rates, or relying on parents to report their children's mixing patterns. A key challenge to collecting such data is obtaining access to the populations of interest and securing the active participation of the subjects, particularly young children. Care must also be taken in the design and interpretation of questionnaires for young children. Young children respond to questioning in a markedly different way from adults [16], with the accuracy of their responses related in a complex way to age, cognitive development, context and the nature of the subject [17]. Several indirect methods have been developed to circumvent these issues, from using mobile phone data [18,19] to using data from websites that exploit a community ethos [20]. In this paper, we present results from a study taking a novel approach in which we used a scientific outreach programme to measure social networks in British primary schools (age 4–11).

We obtained access to our study population through the provision of enrichment material to older secondary school children—effectively using scientific engagement as a research tool. This methodology has allowed us to collect a dataset, which we believe constitutes the most detailed and rich picture of the social mixing patterns of school-age children collected to date. Although our primary motivation has been the measurement of social network measures associated with the risk of disease transmission, we hope that the methodology and dataset will be of significant interest beyond our own field to researchers within the social science and educational communities.

Epidemiologically, schools bring together large numbers of immunologically naive hosts at high density. Schools therefore constitute a core group with the potential to drive the transmission of infectious disease more widely within a community. The classic example of this is the dynamics of childhood diseases, most famously measles, where the timing of the opening and closing of school terms has a demonstrable impact on transmission rates within the community at large [2,21]. However, the importance of schools is not confined purely to diseases of childhood. Children are believed to play a key role in the transmission of acute respiratory illness [22], and in particular influenza [23,24]. School closures [25] and the targeted delivery of vaccine and anti-viral drugs to school children were much discussed during the recent H1N1 pandemic [26–28], with the former carried out in Mexico and Hong Kong [29,30].

Predictions for the efficacy of such interventions vary widely between different theoretical models [3,5–7], in part owing to our lack of understanding of social patterns of mixing that underlie the patterns of transmission we see in epidemiological data. A similar issue arises in the estimation of the basic reproductive ratio, *R*_0_, from serological data. *R*_0_, defined as the expected number of secondary cases in a fully susceptible population resulting from the introduction of a single infectious individual, can in principle be inferred from the age profile of susceptibility for endemic diseases [1]. However, in the absence of quantitative information on the structure of mixing between different age groups, the same serological data can support estimates of *R*_0_ that differ by several orders of magnitude [4,31]. Thus, a key piece of missing information is a detailed quantitative description of how different age groups interact.

Recent large-scale questionnaire studies have provided the first quantitative data that begin to address these problems at a population level [9]. Here, we focus on the key epidemiological unit of primary schools. We apply techniques of social network analysis [10,11,32], with a particular emphasis on estimating how mixing differs within and between different classrooms in the schools. Network methods have a long history of use in sociology and epidemiology [32,33]. Epidemiologically, networks have been particularly well used in understanding the spread of sexually transmitted infections [34,35], but they have also been applied to situations as diverse as tuberculosis [36], influenza [37], foot and mouth disease [38] and obesity [39]. Networks can be used to visualize the connections within a population; they help identify interactions and individuals key to the spread of infection; and they highlight behavioural heterogeneities and patterns of mixing between different population subgroups. High levels of social clustering within networks can slow the spread of infection, with implications for viral evolution [40]. Detailed network data are time-consuming to collect, but as studies continue to probe into the details of social interactions a greater amount of detail is emerging about the structure of human mixing patterns.

## 2. Scientific outreach as a research tool

Recruitment of schools took place in association with the established Motivate Project, part of the Millennium Mathematics Project at the University of Cambridge. Motivate runs a mathematical enrichment programme for schools, enhancing the mathematics curriculum through video conferences between researchers and schools. This approach allows researchers to interact with several schools simultaneously, and enables schools to share ideas quickly and easily. In the project described here, we worked with two groups of secondary schools over two academic years (2007–2008 and 2008–2009).

We developed a series of videoconferences, taking place over the course of an academic year, to share with schools the use of mathematics in understanding disease spread, and the methods and ethics of data collection and analysis. These video conferences allowed school pupils to explore simple epidemic models and to learn about some of the challenges involved in epidemic modelling. Central themes of these video conferences were the importance of primary school children in infection spread and the need for accurate social mixing data.

Over the course of the video conferences, the research team worked with the secondary school students to design a questionnaire that could be used to measure social mixing patterns in primary schools. The brief was to produce a questionnaire that could collect useful information and which was sufficiently straightforward that it could quickly be explained even to the youngest primary school children, the results of which would be discussed and analysed in further video conferences. Ideas were brought together over the course of several video conferences before a questionnaire design was finalized by the research team and consistently applied by all of the schools. The final questionnaire is included as electronic supplementary material.

### (a) Structure of the questionnaire

The questionnaire asked primary school pupils a small number of questions about themselves (age, gender), their household (number and age of siblings) and their social contacts. Here, we present an analysis of the within-school mixing patterns collected through the survey, focusing in particular on changes with age and differences between boys and girls. This network analysis is based on the children's reported social contacts in answer to two key questions:

— ‘Which pupils in your class do you spend the most time with?’
— ‘Which pupils in other classes in the school do you spend the most time with?’

Students were asked to name up to six within-class contacts and up to four outside-class contacts.

### (b) Recruitment of the study population

Six British secondary school groups (age 13–15) took part in the project over two academic years (2007–2008 and 2008–2009). The secondary school groups recruited local primary schools to be surveyed using schools with which they already had established links. Primary schools were therefore recruited on the basis of convenience, in terms of access and proximity to the participating secondary schools, rather than constituting a random selection. Likewise, recruitment of secondary schools was dependent on application by the school rather than selection by the research team. However, the use of video conferencing technology and a track record in recruiting schools to take part in mathematical enrichment events provided by the Motivate Project (one of the programmes that comprise the Millennium Mathematics Project, based on the Mathematics and Education Faculties of the University of Cambridge) enabled the recruitment of schools over a large geographical area and from a range of different communities. Participating schools ranged from small towns in rural Wales to inner-city schools in London and the north of England.

The secondary school groups taking part in this project obtained permission from head teachers to carry out the study in their local ‘feeder’ primary schools. Each secondary school group had to fit the project around the constraints of their normal school curriculum, so the time available was limited. The secondary school students were encouraged to involve one or more local primary schools and to recruit, within those primary schools, a sample of classes that covered a range of year groups. Some larger primary schools contained several classes within each year group; in such cases, secondary schools were encouraged to recruit at least one class from each year group to take part. Informed consent was obtained from the parents of all primary and secondary pupils involved in the study, and from the pupils themselves. Video conferences were also held with many of the primary school children participating, both as science outreach and to explain the potential importance of the data they were being asked to provide. All data were anonymized prior to analysis.

The secondary school participants recruited a total of 11 primary schools, visited each school to explain the project to the primary school children and helped them to complete the questionnaires. A total of 1685 completed questionnaires were received from these primary schools, representing an 89.2 per cent recruitment rate, defined as the fraction of those primary school pupils whose classes were recruited that returned the questionnaires. Missing data were generally as a result of absence from school on the day the study took place.

## 3. Results

**Figure 1.**
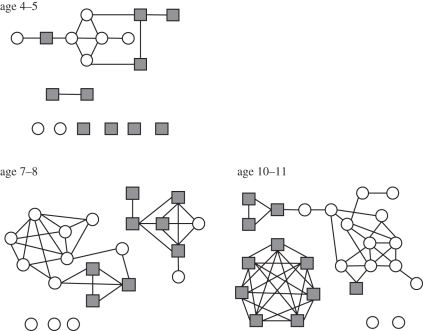
Sample networks of self-reported social contacts within a classroom. Networks of mutually reported social contacts for three representative classrooms taken from the same primary school demonstrating increasing complexity, cliquishness and gender segregation with age. Filled squares, girls; open circles, boys.

Figure 1 shows sample networks collected from three classes within the same primary school, consisting of children in the age ranges of 4–5, 7–8 and 10–11. We plot a link between two individuals only if each names the other (so-called ‘mutual links’). The data collected could be used to generate directed networks. However, these can be challenging to visualize clearly and are likely to be more susceptible to biases in the reporting of contacts (an issue we shall discuss further below). Figure 1 displays several striking properties that we shall consider in turn: a large number of mutual links, segregation between boys and girls, and a high level of social clustering.

### (a) Mutual links

A central issue with the interpretation of self-reported social contacts is quantifying the extent to which individuals can accurately identify their social contacts. The ability of young children to provide accurate information under questioning has received a great deal of attention, in particular with respect to assessing the validity of children's testimony in court cases [41]. On interview, young children often demonstrate a susceptibility to suggestion [41] and a tendency to manufacture responses to ‘unanswerable’ questions [42]. The forms of the questions posed are particularly important. Young children are less likely to provide fanciful answers to ‘open’ questions rather than those that are ‘closed’ categorically (yes/no) [42]. For simple objective questions, children are potentially capable of achieving similar levels of accuracy to adults [43], particularly with respect to objects or events that they have a particular interest in [41].

As previously described, rather than specifying a fixed number of choices or selecting contacts from a list, students were asked to name up to six within-class contacts and up to four outside-class contacts. If the true number of contacts exceeds these enforced limits, then the reported contacts will be censored. However, imposing no limit on the number of contacts introduces the risk that peer pressure may result in ‘competitive naming’ of contacts in order to appear more popular. There is also the possibility that ‘closing’ the question in this way may have encouraged respondents to provide additional spurious names beyond those they would subjectively rank as their *closest* contacts. The final design therefore sought to achieve a compromise between limiting the impact of these two potential sources of bias: censoring and over-reporting.

**Figure 2.**
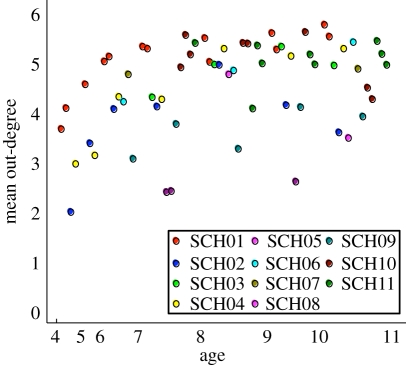
Mean out-degree (number of within-class contacts named per person) against age of school class. Data are plotted for each class in the survey, with the colour representing the school. The sample contains several classes within each age group, and the ordering of points within age groups is not significant: all classes plotted between ages 4 and 5, for example, have pupils aged between 4 and 5.

Plotting the out-degree by class suggests a trend towards older respondents reporting more contacts (figure 2). However, there is considerably more variation between classes than with respect to age. Because of the potential bias introduced by censoring, these mean outdegrees should be considered as a lower bound on the true values. Variation in the interpretation of the questionnaire questions by (primary school) respondents and the emphasis placed by different (secondary school) interviewers may have also led to biases in the criteria by which individuals prioritize their social contacts. For example, children who are perceived to be popular might be expected to attract links through aspiration of the responder rather than owing to an established relationship.

**Figure 3.**
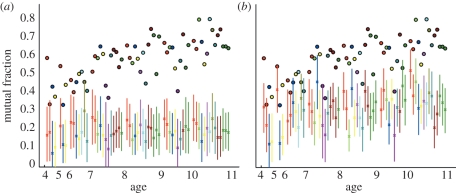
Fraction of mutual links against age of school class. A comparison of the data (circles) with the output of simulated networks of contacts within each class (mean (crosses) and central 99 percentiles (lines)). Colour scheme as in figure 2. (*a*) Simulated networks contain the observed number of individuals each with their observed out-degree. (*b*) Simulated networks include gender information; each contains the observed number of individuals with their observed number of contacts with boys and with girls (see Appendix for further details on generation of simulated networks).

We attempt to control for, and quantify, these biases by considering *mutual* links, where pairs of individuals identify each other as social contacts [11]. It would be natural to expect that reciprocated links are more representative of *relationships* rather than *popularity*. Overall, 61 per cent of contacts in the data are mutual. The fraction of mutual links increases with the age of the respondent (figure 3). Even for the youngest age groups, the fraction of mutual links is significantly greater than we would expect to see purely by chance (figure 3*a*), suggesting that the reported contact networks are indeed evidence of established social structure.

### (b) Gender assortativity

**Figure 4.**
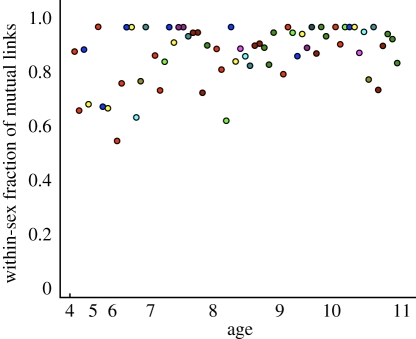
Gender assortativity against age of school class. The fraction of mutual links that connect individuals of the same gender. Colour scheme as in figure 2.

The majority of the within-class networks display a clear segregation of boys and girls according to the reported social contacts—indeed, in several classes there were no reported mutual links between girls and boys. The segregation becomes more striking for the older age groups (figure 4). This observed gender segregation contributes to the high number of mutual links, but even when taking the patterns of gender-based mixing into account there are significantly more mutual links observed than would be expected (figure 3*b*). This gender assortativity is therefore not sufficient to explain the excess number of mutual links, indicating once more that there is additional social structure in the reported contacts.

### (c) Social clustering

**Figure 5.**
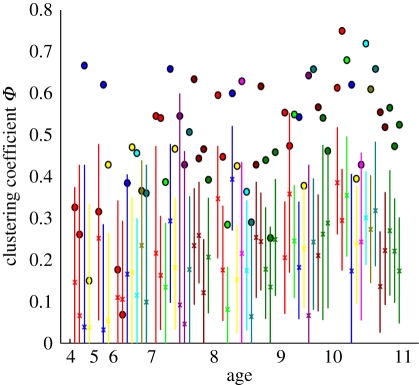
Clustering against age of school class. Clustering within the network of mutual links, measured by the clustering coefficient *ϕ*. A comparison of the data (circles) with the output of simulated networks of contacts within each class (mean (crosses) and central 99 percentiles (lines)). Colour scheme as in figure 2. Simulated networks contain the observed number of mutual links and the observed patterns of gender mixing (see Appendix for further details on generation of simulated networks).

Social clustering in within-class networks was measured by the clustering coefficient *ϕ*, defined as the number of triples divided by the number of triangles (or, more simply, ‘the probability that two of my contacts know each other’). *ϕ* is observed to increase with age (figures 1 and 5). Given the high proportion of mutual links and gender assortativity, it is perhaps unsurprising that the within-class networks exhibit large numbers of ‘cliques’ (figure 1). To explore the extent to which clustering is driven by these known factors, we generated distributions of the clustering coefficient, *ϕ*, from random networks containing the same number of mutual links and the same patterns of gender-based mixing [33]. For the majority of our within-class networks, *ϕ* is significantly greater than is seen in these simulated networks (figure 5), suggesting that even these strong observed heterogeneities are not sufficient to explain the observed social structures.

### (d) Contacts between age groups

**Figure 6.**
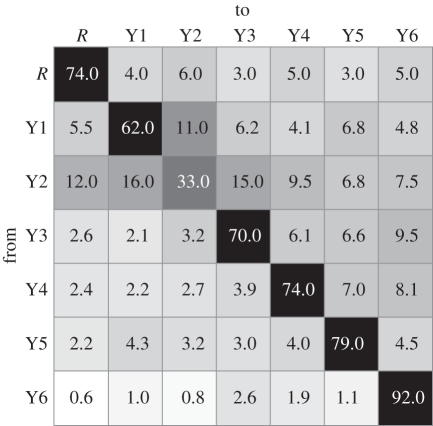
Reported contacts between cohorts. Reported contacts between different classes were classified according to the year group (*R* = reception, Y1 = year 1, etc.) of the student filling in the questionnaire (‘from’) and the year group of the named contact (‘to’). These were normalized by the total number of reported contacts from each year group and are presented here as inset percentages; darker shading corresponds to a higher percentage. The data presented here are limited to the schools for which each year has multiple classes. For all years, the majority of reported contacts external to the respondents' own class are nonetheless within the same school year. Reported contacts are asymmetric, with a greater proportion of reported contacts directed to year groups senior to the respondent.

Finally we consider the patterns of mixing between different classrooms. Because of the limits placed on the number of contacts listed, we cannot compare the relative proportions of within-class contacts to between-class contacts. However, in schools with more than one class in each year group, we can compare the fraction of reported between-class contacts that are within-year and between-year. Mixing between age groups is strongly assortative, with approximately 80 per cent of between-class contacts being reported within age group (figure 6).

## 4. Discussion

We have successfully applied simple questionnaire-based methods to measure social networks within complete primary school classes, and in some cases complete schools, in Britain. The networks of reported social contacts demonstrate a great deal of complex structure, with important implications for the spread of infectious diseases and the interpretation of epidemiological data. There is a striking segregation between the genders, with very little reported social communication between boys and girls, and tight, internally connected cliques that become more evident in older groups. Sociological studies have consistently found similar patterns of sex bias and clustering in school peer networks [10–15]. However, no study to our knowledge has attempted to survey exhaustively entire school populations or considered the implications of detailed school mixing patterns for disease transmission.

Interpreting social contact networks in an epidemiological context can be delicate as there is no clear definition of what is likely to constitute an ‘infectious contact’. The reported social contacts within our study are based on the qualitative judgement of the respondent as to ‘which pupils … you spend the most time with’. We chose our question based on the assumption that the contacts children spend most time with are those for which there is the greatest potential for the transmission of disease. It would be natural to expect that a child's reported contact may vary depending on the semantic phrasing of this question. The fact that such a large number of the contacts were mutual links suggest that there was a high level of consistency in the interpretation of the question.

Not all primary school students could complete the questionnaire with equal ease; older primary school children were generally able to fill out the questionnaire with minimal assistance, but the youngest age groups required more individual attention from the secondary school students. By confining our analysis to mutually reported contacts, we hope to limit the potential for variations in the implementation of the questionnaire and subsequent interpretation by the respondents to bias our results. However, we must keep these subjective issues in mind when interpreting these data. For example, we cannot distinguish whether the increase in the fraction of mutual links with age is evidence of an increase in social structure or in the accuracy of respondents' ability to accurately report their social contacts. The reported number of contacts between age groups suggests that there are some age-dependent biases in reporting. Overall, children within the study were more likely to report contacts in older year groups rather than younger (figure 6). The question as to whether this was because of an aspirational selection of ‘older’ contacts (which was not reciprocated) or merely a function of the age-dependent accuracy of self-reported contacts would be an intriguing avenue for further study.

The phrasing of our question is subtly distinct from those previously used in social network studies in schools. From a sociological perspective, the duration of contacts is less interesting than understanding the nature of relationships and their formation [14]. A more typical question posed by previous studies would therefore be of the form of ‘who would you *like* to spend the most time with’ or ‘name your *best* friend’. Although we have set out to measure a different aspect of children's social relationships, we acknowledge that a child's perception of whom they ‘spend the most time with’ may well be intermingled with who they ‘would like to spend the most time with’ [14].

These semantic issues could be addressed through gathering more detailed information than we have attempted here. For instance, respondents could be asked to classify the type of contacts in a ‘contact diary’. Given our study population of mostly young children, adding extra levels of complexity to a survey would not necessarily meet with success. Ideally, it would be desirable to eliminate the subjective nature of these questions. Electronic methods of measuring social contact networks offer the potential to overcome this problem [44]. Furthermore, electronic methods could be used to quantify the structure of chance contacts on the playing fields and in the dining halls and corridors, which are likely to form an important role in disease transmission but are far less straightforward to capture through questionnaire methods. However, given the privacy issues associated with such intensive surveillance and the vulnerable nature of our study population, it is not certain that such methods would be acceptable to schools, parents or children, even though it may well form a fruitful area for further research.

The notion of a ‘risky contact’ varies greatly between different infectious agents. For short, acute respiratory infections, we might expect a classroom to constitute a single homogenously mixed unit even in the presence of the strong patterns of non-random mixing seen within these data. For such infections, therefore, it is the mixing between age groups that is more interesting and has not been studied to the same extent as within-class social networks. Our data verifies the long-held belief that mixing is strongly assortative between the age-groups in a school [1,45] on a finer scale than has been previously demonstrated [9].

These age-related mixing patterns are essential for the proper interpretation of epidemiological data for childhood infectious diseases such as measles, mumps and rubella, which are typified by a long-lasting immunity to reinfection after recovery. This is most easily characterized through the fundamental epidemiological concept of the basic reproductive ratio, *R*_0_ [1]. Defined as the expected number of new infections on the introduction of a single infective agent into a fully susceptible population, *R*_0_ sets the upper bound on the reproductive potential of a pathogen and, conversely, the effort required to bring it under control. The presence of immunity within a population serves to limit the rate of spread within a population, leading to a net, or effective, reproductive ratio (*R*) [1].

It is relatively straightforward to estimate *R* from serological data [4] or time series of case reports [2] set against the current background level of susceptibility within the community. However, if transmission rates are heterogeneous with respect to age, then the estimated value of *R*_0_ depends critically on the architecture of the contacts between different age groups [4]. This has traditionally been accounted for by defining symmetrical matrices that specify the form of transmission between age groups, styled as ‘who aquires infection from whom’ matrices [1]. In general, the more assortative that contacts are with respect to age, the larger the corresponding value for *R*_0_ will be, based on the same value of *R* [31]. Our data provide a quantitative basis to support the selection of contact matrices and thus, hopefully, to improve predictions for *R*_0_ for this important class of human diseases.

For infections transmitted through close social contacts, the clustering of individuals' contacts within the classroom is likely to become more important. Once again, the importance of these patterns lies in the interpretation of epidemiological data rather than in general terms of the rate of spread of an infection. An obvious example for further study would be the prevalence of head-lice within schools, which are notably found at higher rates in girls when compared with boys [46]. Given that intimacy is likely to aid the transmission of head-lice, the pronounced sex bias in reported contacts reported here might be relevant to understanding the transmission dynamics of ectoparasites in a school classroom. However, the signature for this would lie in the variability of the sex bias and the persistence of disease between different outbreaks rather than in a systematic bias.

The data obtained by this project required the investment of significant time and effort, on behalf of both the research team and the secondary school participants and their teachers. Indeed, this project was only possible owing to the involvement of large numbers of secondary school students in the motivation, design and implementation of the study. In itself, this may be considered to be a major limitation of the study. School children cannot be expected to rigorously apply a questionnaire or, in the case of the youngest primary school children, perform the same structured interview technique for every respondent. This introduces the possibility that variations between the different secondary school groups involved in the project may have led to unquantifiable biases in the collected data.

However, during the development of the project it became clear that performing the study in collaboration with secondary school students contributed several major advantages over more traditional ‘researcher-driven’ methodologies. The most important was simply the access to the study populations themselves. Surveying children is particularly difficult, as consent must be obtained from schools and families, and time taken to explain the methodology to teachers and parents. Recruiting our study populations indirectly through a public engagement project with secondary school children greatly facilitated this process. The existing links between the secondary schools and their feeder primary schools were the essential key to providing access. Both secondary and primary schools were keen to develop these links in an innovative way, which was further enhanced through the provision of on-site visits from the research team for some of the participating schools. The pre-existing videoconferencing infrastructure provided through Motivate allowed us to brief the teachers directly, who in turn provided the logistical support to deliver and process consent forms for the study. In return, the research team provided enrichment to the national maths and science curricula for the older children, and the experience of working on a genuine research project with a prestigious university.

The secondary students provided an important source of ‘local knowledge’ for the design and execution of the study. In many cases, the students revisited the primary schools that they themselves had attended, so they were acquainted with the school structure and culture before embarking on the data collection. Being closer in age and background to the respondents was also beneficial for establishing a rapport with the younger children who required more individual attention. This was a particular issue for some of the inner-city schools where children came from a variety of cultural and linguistic backgrounds.

The combined distributed effort of all of the secondary school participants has provided a dataset of social networks in schools that is unrivalled in its scope, size and detail. By combining our survey method with public engagement, we have exhaustively sampled 75 complete primary school classes from 11 primary schools in Britain with a high response rate (around 90% in most schools). The unique aspect to this approach is that part of the study population themselves—in the form of the secondary school participants—were involved in the planning, collation and analysis of the data. Epidemiology is a research area that directly concerns and affects the public. Mathematical models are becoming an ever more important part of the development of public health policies for the control of emerging infectious diseases. Explaining the mechanisms and assumptions inherent in these approaches to the public at large is vital if they are to make informed decisions about these important issues. In this project, we went further and used public engagement as a *methodology* to perform our research, in the process helping to inspire the next generation of epidemiologists and mathematical modellers. The work carried out by these engaged and motivated secondary school pupils was essential to the project, without which it could not have been a success.

## Appendix

Materials developed during the project are available at http://motivate.maths.org/content/DiseaseDynamics.

### (a) Social network analysis

The results of the questionnaire were described using methods of social network analysis. An adjacency matrix *A* was calculated for each participating class, such that *A*(*i*,*j*) = 1 if person *i* named person *j* as a contact and *A*(*i*,*j*) = 0 otherwise. Networks of contacts were plotted, allowing straightforward identification of heterogeneities within the network; outdegrees and in-degrees were calculated for each individual. The fraction of mutual links was calculated as ; the restriction in the sum in the denominator exists because a mutual link can only be observed between *i* and *j* if both individuals completed the survey. Within the networks, clustering was measured using the clustering coefficient, *ϕ*, defined as the number of triples divided by the number of triangles (or, more simply, ‘the probability that two of my contacts know each other’).

To explore the extent to which the measured networks differed from random graphs, simulated networks were generated. These simulated networks contained links placed at random, subject to certain constraints. When looking at the number of mutual links (figure 3*a*), the simulated network matched each node's observed out-degree, and, when considering gender mixing (figure 3*b*), each node's number of contacts with boys and with girls. When considering clustering (figure 5), the simulated networks contained the observed number of mutual links both within and between genders—in this instance, simulated networks contained only individuals who completed the survey (since non-responders could not, by definition, have any mutual links). Simulated networks were built link by link. When matching mutual links, pairs of individuals were picked at random and, when matching to out-degree, potential contacts were chosen at random for each individual one by one. In both cases, proposed links were only accepted provided that they did not violate the properties of the given data. Measures of network statistics—number of mutual links and clustering coefficient—were obtained from these simulated networks and compared with the values observed in the data. For each class and each comparison, 1000 simulated networks were generated.

### (b) Study population

The complete dataset of reported contacts is presented as electronic supplementary material to this paper. Tables S1–S12 in the electronic supplementary material summarize this data in terms of the range, size and response rates for the schools and classes sampled over the 2 years of the project. A small number of mixed year group classes (one aged 5–7, two aged 7–9, three aged 9–11) were sampled, and these have been classified within the upper categories in the electronic supplementary material, tables S1 and S2. Some classes, particularly in schools 5, 7 and 8, were unusually small or poorly sampled. Six classes with fewer than 16 pupils and six classes with a response rate of lower than 75 per cent have been considered as outliers and omitted for the analyses presented in this paper; these 12 classes are spread across all age groups, with one aged 4–5, three aged 5–6, one aged 6–7, three aged 7–8, one aged 8–9 and three aged 9–10. These outliers are identified in tables S3–S12 of the electronic supplementary material. Versions of figures 2–5 including these outliers are also presented in the electronic supplementary material. Excluding the 12 outliers reduces the sample size to 1521 pupils, with a response rate of 92.4 per cent.
